# Treatment outcomes of COVID-19 infection in PSYCHIATRIC HOSPITAL

**DOI:** 10.1192/j.eurpsy.2022.527

**Published:** 2022-09-01

**Authors:** V. Borkovic, K. Župan, D. Šuša

**Affiliations:** Psychiatryc Hospital Ugljan, Geriatrics, Ugljan, Croatia

**Keywords:** Covid-19, psychiatry patients

## Abstract

**Introduction:**

The ongoing pandemic of coronavirus disease 2019 (COVID-19) has made a serious public threat worldwide. The first case in the Republic Croatia was reported on 25^th^ February 2020 and the first case in Psychiatric hospital Ugljan was diagnosed on 3^rd^ December 2020. To maximize protection and prevent spreading to other patients, COVID-infected-individuals were isolated. This poster will describe treatment outcomes of COVID 19 in Psychiatric hospital Ugljan.

**Objectives:**

This rapid review summarizes outcomes of COVID-19 infected psychiatric patients with mild disease severity to provide synthesized evidence to support policy decision making.

**Methods:**

PubMed, Medline, PsychINFO were systematically searched from January 2021 for COVID-19, with studies describing epidemiology, treatment and outcomes in various long-term care facilities. Studies were excluded if they did not report clinical evidence.

**Results:**

In the time of COVID-19 breakthrough in psychiatric hospital, in December 2020 328 patients were hospitalized of whom 44 tested positive for SARS-CoV-2 infection: of 307 hospitalized patients in January 2021 36 tested positive for SARS CoV-2. By that time, there were no treatment options available, so we focused on repurposing efficacy of the currently used drugs. Five patients needed admission to ICU, we reported one death from coronavirus disease 2019 in that period.

**Conclusions:**

The COViD-19 pandemic has highlighted extreme vulnerability of psychiatric patients who reside in long-term care psychiatric hospitals and there is an urgent need for evidence-based policy that can protect adequately psychiatric patients.

**Disclosure:**

No significant relationships.

